# Pyrrolidine-Based Antifungal Agents Against *Candida* Species: A Systematic Review of Structure–Activity Relationships and Mechanistic Insights from Experimental Studies

**DOI:** 10.3390/ph19081198

**Published:** 2026-07-31

**Authors:** Harinahalli Ganesh Sinchan, Nagaraju Chaithra, Shivaswamy Umamaheshwari

**Affiliations:** 1 Department of Microbiology, School of Life Sciences, JSS Academy of Higher Education & Research, Mysuru 570015, Karnataka, India; sinchanhg1296@gmail.com; 2 Division of Medical Statistics, School of Life Sciences, JSS Academy of Higher Education & Research, Mysuru 570015, Karnataka, India; chaithra.mstats@jssuni.edu.in

**Keywords:** pyrrolidine derivatives, *Candida* species, antifungal activity, biofilm inhibition, structure–activity relationship, systematic review

## Abstract

**Background:** Invasive and mucosal infections caused by *Candida* species continue to pose a significant global health burden, particularly due to the species’ increasing resistance to conventional antifungal agents. This systematic review evaluates the antifungal potential of pyrrolidine-based derivatives, focusing on structure–activity relationships, mechanisms of action, and activity against *Candida* species. **Methods:** A systematic literature search was conducted in PubMed, Scopus, and Web of Science for studies published between January 2015 and January 2026, following PRISMA guidelines and registered in PROSPERO (CRD420261279035). Studies reporting in vitro, in vivo, or in silico evaluation of pyrrolidine derivatives against *Candida* species were included. Data extraction and risk-of-bias assessment were performed using predefined criteria. **Results:** Twelve studies met the inclusion criteria, describing diverse pyrrolidine-based scaffolds, including spirooxindole, dispiro, triazole-linked, tetrazole, and quinoline-fused derivatives. The compounds demonstrated antifungal activity against multiple *Candida* species, including resistant isolates with minimum inhibitory concentrations ranging from approximately 0.5 to 16 µg/mL. Several derivatives showed comparable activity to standard antifungal agents under the reported experimental conditions. Mechanistic investigations indicated inhibition of biofilm formation, disruption of fungal cell wall and membrane integrity, suppression of hyphal transition, and modulation of virulence-associated pathways. Multi-mechanism antifungal effects were reported across different structural classes. **Conclusions:** Pyrrolidine-based derivatives represent promising antifungal scaffolds with potent activity against *Candida* species and associated virulence factors. However, limited in vivo validation and insufficient pharmacokinetic and toxicity data remain major barriers to clinical translation. Further optimization of structure–activity relationships and comprehensive preclinical evaluation are required to advance these scaffolds toward therapeutic development.

## 1. Introduction

Invasive candidiasis remains a significant global public healthcare concern, particularly among immunocompromised individuals, patients receiving prolonged antibiotic therapy, and those with indwelling medical devices. *Candida* species are responsible for a broad spectrum of infections ranging from superficial mucosal disease to life-threatening systemic infections, resulting in substantial morbidity and mortality worldwide [1,2]. In recent years, the emergence and dissemination of multidrug-resistant non-*albicans Candida* species such as *Candida auris, Candida glabrata*, and *Candida tropicalis* have further complicated disease management, highlighting the urgent need for new therapeutic strategies [3,4].

Currently, antifungal therapy relies primarily on three major drug classes: azoles, echinocandins, and polyenes [5,6]. Despite the clinical relevance of these drugs, the effectiveness of their prolonged use is increasingly limited by antifungal drug resistance, toxicity, and reduced efficacy against biofilm-associated infections [7,8]. Biofilm formation represents a critical virulence trait of *Candida* species, providing protection against host immune responses and substantially reducing susceptibility to conventional antifungal agents [9,10].

Consequently, the discovery of small molecules with novel mechanisms of antifungal action has become a research priority [11,12]. Among the diverse classes of heterocyclic compounds explored for antifungal drug discovery, pyrrolidine derivatives have attracted considerable attention owing to their structural versatility, stereochemical complexity, and favourable interactions with biological targets [13,14].

Previous studies have demonstrated that pyrrolidine-based compounds exhibit significant antifungal activity against *Candida* species through multiple mechanisms, including disruption of membrane integrity, inhibition of biofilm formation, suppression of ergosterol and chitin biosynthesis, modulation of efflux pump activity, and interference with virulence-associated genes [15,16,17]. Moreover, structural modification strategies including spirocyclization and addition of heterocyclic pharmacophores (such as triazoles, quinolines, and tetrazoles) have resulted in improved antifungal activity against various drug-resistant fungi [17,18,19].

Despite growing evidence supporting the antifungal potential of pyrrolidine derivatives, important knowledge gaps remain regarding their mechanisms of action, pharmacokinetics, and toxicological profiles. Furthermore, most published studies are limited to in vitro and in silico investigations, with relatively few in vivo evaluations or comprehensive pharmacokinetic and toxicity assessments [12,20]. Considerable heterogeneity in experimental methodologies, fungal strains, and outcome measures further limits direct comparison across studies [19,21].

To date, no systematic review has comprehensively synthesized the available evidence on pyrrolidine-based compounds with anticandidal activity. This review addresses the gap by critically integrating evidence on structure–activity relationships, mechanisms of action and antifungal efficacy against *Candida* species. It also identifies promising pyrrolidine scaffolds, highlights key research gaps, and outlines priorities for the rational development of next-generation antifungal agents.

## 2. Materials and Methods

### 2.1. Protocol and Reporting Guidelines

This systematic review was conducted in accordance with the Preferred Reporting Items for Systematic Reviews and Meta-Analyses (PRISMA) guidelines. The review protocol was prospectively registered in the International Prospective Register of Systematic Reviews (PROSPERO) under registration number CRD420261279035. The review methodology, eligibility criteria, and outcomes of interest were defined prior to study selection to minimize reporting bias and enhance methodological transparency.

### 2.2. Eligibility Criteria

Eligibility criteria were defined a priori in accordance with the *Candida*-related/pyrrolidine-related Population, Intervention, Comparator, Outcomes, and Study Design (PICOS) framework to ensure methodological transparency and reproducibility.

#### 2.2.1. Population

Studies investigating *Candida* species such as *Candida albicans*, *Candida auris*, *Candida glabrata*, *Candida tropicalis*, *Candida krusei* and other clinically relevant *Candida* isolates were considered eligible.

#### 2.2.2. Intervention

Studies evaluating pyrrolidine-containing compounds, including pyrrolidine derivatives, spiro-pyrrolidines, dispiropyrrolidines, pyrrolidine-fused heterocycles, and structurally modified pyrrolidine analogues with reported antifungal activity against *Candida* species, were included.

#### 2.2.3. Comparator

Where applicable, studies were included if they used appropriate comparators such as standard antifungal agents (e.g., fluconazole, amphotericin B, caspofungin, voriconazole), untreated controls, or vehicle/solvent controls. Studies without comparator groups were also included if antifungal activity was directly assessed.

#### 2.2.4. Outcomes

Studies were required to report at least one relevant antifungal or mechanistic outcome. Eligible outcomes included, but were not limited to:

- Minimum inhibitory concentration (MIC);
- Minimum fungicidal concentration (MFC);
- Inhibition or eradication of biofilm formation;
- Suppression of hyphal transition or morphological switching;
- Effects on fungal adhesion and virulence factors;
- Cytotoxicity or selectivity indices;
- Mechanistic endpoints such as ergosterol biosynthesis disruption, chitin synthase inhibition, oxidative stress induction, or membrane integrity disruption;
- Computational outcomes such as molecular docking, binding affinity analysis, target prediction, or structure–activity relationship (SAR) modelling when relevant to antifungal mechanisms.

#### 2.2.5. Study Design

Original research articles employing experimental and/or computational methodologies were eligible for inclusion. These comprised:

- In vitro studies assessing antifungal, antibiofilm, or mechanistic activity of pyrrolidine-based compounds against *Candida* species.
- In vivo studies evaluating antifungal efficacy in animal models of candidiasis or fungal infection.
- In silico studies involving molecular docking, pharmacophore modelling, target prediction, or SAR analysis, provided that these contributed mechanistic or binding-level insights relevant to antifungal activity.

Studies employing integrated experimental and computational approaches were also included.

#### 2.2.6. Additional Eligibility Criteria

To ensure relevance and quality, the following restrictions were applied:

- Only peer-reviewed original research articles were included;
- Studies published between January 2015 and January 2026 were considered;
- Only articles published in the English language were included.

#### 2.2.7. Exclusion Criteria

Studies were excluded if they met any of the following criteria:

- Review articles, systematic reviews, meta-analyses, editorials, commentaries, conference abstracts, or book chapters;
- Studies not involving pyrrolidine-based compounds or derivatives;
- Studies not evaluating *Candida* species or antifungal activity relevant to candidiasis;
- Studies lacking primary antifungal, mechanistic, or computational data;
- Duplicate publications or overlapping datasets;
- Non-English-language publications.

### 2.3. Information Sources and Search Strategy

A comprehensive literature search was performed in PubMed, Scopus, and Web of Science databases. The last search was conducted on 15 January 2026. A broad search strategy combining pyrrolidine-related terms and *Candida*-related terms was used to maximize sensitivity while minimizing the risk of missing relevant studies.

The search strategy incorporated both Medical Subject Heading (MeSH) terms and free-text keywords combined with Boolean operators:

- (pyrrolidine OR pyrrolidines OR pyrrolidine-derived OR pyrrolidine-based OR spirooxindole-pyrrolidine OR dispiropyrrolidine OR spiroquinoline-pyrrolidine OR tetrazole-pyrrolidine OR pyrrolidine-triazole) AND (Candida OR candidiasis OR Candida albicans) AND (antifungal OR antifungal activity OR biofilm OR anti-biofilm).
- Database-specific adaptations were applied to optimize sensitivity and retrieval across platforms.

### 2.4. Study Selection

All retrieved records were imported into Microsoft Excel for the removal of duplicate entries. Study selection was performed in two sequential stages:

1. Screening of titles and abstracts was performed to exclude irrelevant studies.
2. Full-text assessment based on predefined eligibility criteria.

Disagreements between reviewers were resolved through discussion or consultation with a third reviewer. The study selection process was documented using a PRISMA flow diagram.

### 2.5. Data Extraction

Data extraction was independently performed by two reviewers (SHG and CN) using a standardized data extraction form. Any disagreements identified during the extraction process were discussed and resolved with the involvement of a third reviewer (US).

Extracted variables included:

- Author and year of publication;
- Geographic location;
- Study design (in vitro, in vivo, in silico);
- Pyrrolidine derivative class and chemical scaffold;
- Target *Candida* species;
- Experimental methodology;
- Antifungal outcomes (MIC, MFC, biofilm inhibition);
- Proposed mechanisms of action.

### 2.6. Data Synthesis

A qualitative synthesis was performed due to heterogeneity in experimental models, tested compounds, and outcome measures across included studies. No meta-analysis was conducted. Results were grouped according to structural subclasses of pyrrolidine derivatives and their corresponding antifungal mechanisms of action.

### 2.7. Quality Assessment

The quality assessment included the following domains: (i) clarity of study objectives, (ii) appropriateness and description of study design, (iii) clarity of defined selection criteria, (iv) reporting of inclusion and exclusion criteria, (v) identification and characterization of pyrrolidine derivatives, (vi) clarity of description of target organisms, (vii) adequacy of experimental methodology, (viii) inclusion of appropriate control groups, and (ix) clarity of reported outcomes. Each criterion was scored as either 1 (criterion met) or 0 (criterion not met) based on the completeness of reporting and methodological transparency, yielding a maximum possible score of 9 for each study.

Based on total score, studies were classified as:

- 0–5= low-quality;
- 6–7 = moderate-quality;
- 8–9 = high-quality.

Disagreements between the reviewers were resolved through discussion, and when required, a third reviewer was consulted to achieve consensus. Studies demonstrating adequate methodological reporting across most domains were considered to be of moderate to high quality. 

### 2.8. Risk of Bias

The methodological quality of included studies was assessed using a structured risk-of-bias framework adapted from Cochrane Collaboration guidelines and SYRCLE (Systematic Review Centre for Laboratory Animal Experimentation) recommendations for preclinical studies.

The following domains were evaluated:

- Selection bias;
- Performance bias;
- Detection bias;
- Reporting bias.

## 3. Results

### 3.1. Study Selection

**Figure 1:**
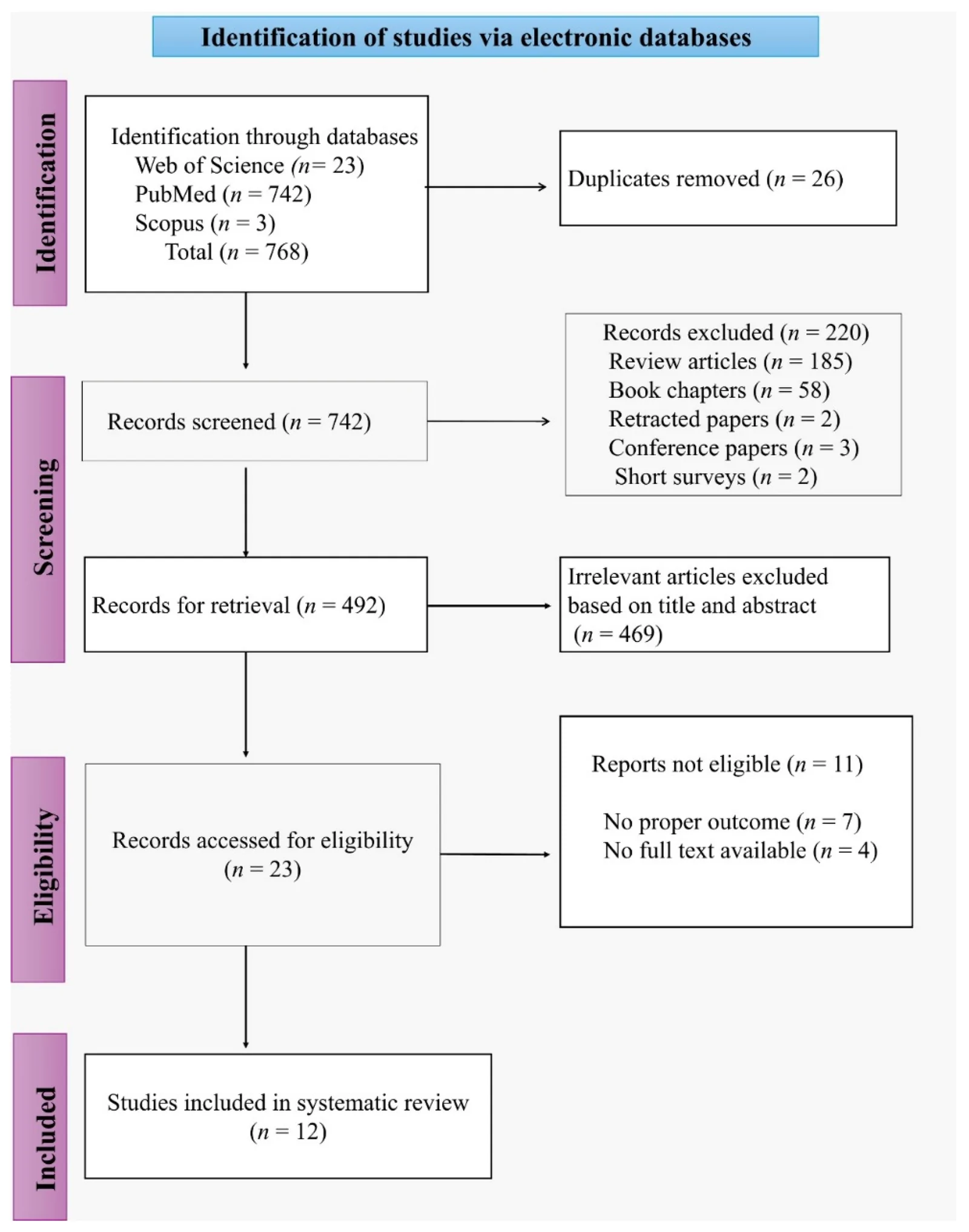
Flowchart of study selection process.

A total of 768 records were retrieved from PubMed (*n* = 742), Web of Science (*n* = 23), and Scopus (*n* = 3). After removal of duplicates across all databases, records were screened for eligibility. Following title and abstract screening, 23 full-text articles were assessed for inclusion. Finally, 12 studies met the predefined eligibility criteria and were included in the systematic review. The study selection process is illustrated in the PRISMA flow diagram (Figure 1).

### 3.2. Study Characteristics

The final analysis included 12 studies published during the review period. Most studies were conducted in vitro, whereas only a limited number included in vivo experiments or in silico analyses investigating pyrrolidine-based derivatives against *Candida* species.

The included studies originated from diverse geographical regions, including the United States, China, India, France, Romania, Italy, Poland, Saudi Arabia, Tunisia, and South Africa, reflecting global research interest in pyrrolidine-based antifungal scaffolds.

A wide variety of pyrrolidine-containing compounds was reported. The major scaffold classes included spirooxindole–pyrrolidine hybrids, dispiro systems, pyrrolidine–triazole derivatives, spiroquinoline–pyrrolidine frameworks, tetrazole-substituted pyrrolidines, hydrazone-linked pyrrolidones, and fused heterocyclic systems incorporating pyrrolidine cores.

Among the included studies, spiro-fused pyrrolidine derivatives were the most extensively studied scaffold class. Antifungal activity was evaluated predominantly against *Candida albicans*, although several studies also examined clinically important non-albicans *Candida* species, including *Candida auris, Candida glabrata*, and *Candida tropicalis*.

The reported antifungal potency varied according to scaffold architecture and substitution pattern, with minimum inhibitory concentration (MIC) values ranging from ≤1 µg/mL for the most active compounds to approximately 16 µg/mL for moderately active derivatives. Several pyrrolidine-based compounds exhibited antifungal activity comparable to that of established antifungal agents, including fluconazole and amphotericin B.

Beyond growth inhibition, many studies reported additional anti-virulence effects, such as suppression of biofilm formation, inhibition of hyphal development, and reduced fungal adhesion. However, assessments of cytotoxicity, selectivity, and pharmacological safety were performed in only a limited number of studies. Similarly, in vivo validation remained scarce, restricting conclusions regarding clinical applicability.

Despite the promising findings, substantial heterogeneity was observed across studies with respect to fungal strains, experimental models, susceptibility testing methods, and outcome reporting. Consequently, quantitative meta-analysis was not feasible, and the results were synthesized using a narrative approach (Table 1).

### 3.3. Antifungal Activity and Structural Diversity

All included compounds exhibited antifungal activity against *Candida* species, with most studies focusing on *Candida albicans* and emerging resistant species such as *Candida auris*. Minimum inhibitory concentration values varied depending on structural class and substitution pattern. Potent compounds demonstrated MIC values in the range of 0.5–1 µg/mL, while moderate activity was generally observed between 4 and 16 µg/mL. Several compounds demonstrated activity comparable to or exceeding that of standard antifungal agents such as fluconazole, particularly against resistant isolates (Table 2).

Antibiofilm activity was reported in the majority of included studies. Inhibition of biofilm formation, hyphal transition, and cell adhesion was consistently observed across spirooxindole and dispiro derivatives. In several cases, the tested compounds demonstrated superior biofilm inhibition compared with clinically used antifungal agents, highlighting their anti-virulence potential.

### 3.4. Mechanistic Insights

Mechanistic evaluation across included studies indicates that pyrrolidine-based derivatives exert antifungal effects through multiple pathways. However, the level of evidence varied among studies depending on whether the findings were derived from experimental assays or computational predictions.

**Figure 2:**
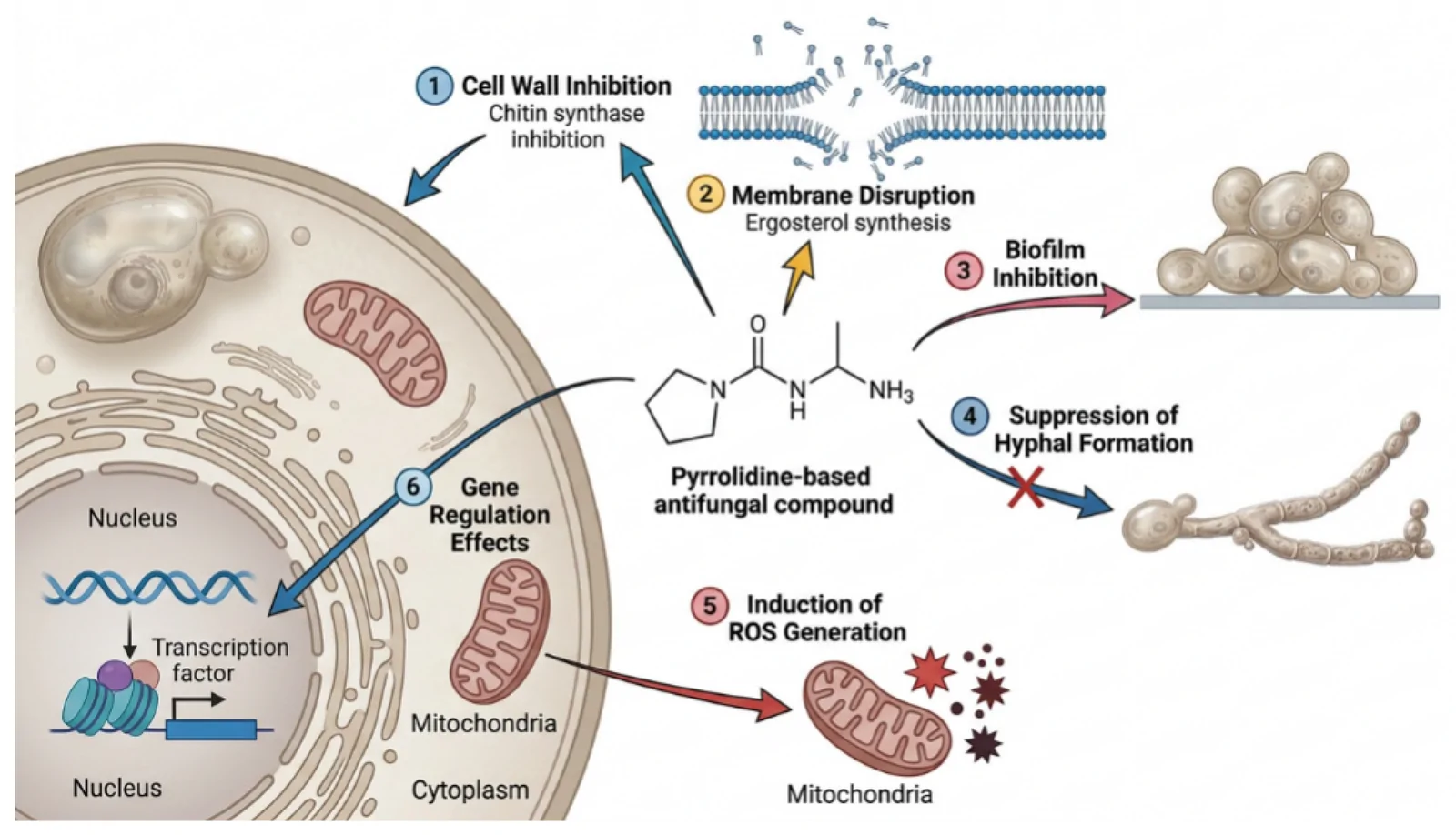
Summary of proposed antifungal mechanisms of pyrrolidine-based derivatives against *Candida* species.

Disruption of fungal cell wall integrity was the most frequently reported mechanism via inhibition of chitin synthase activity. This was supported by sorbitol protection assays in several spiroquinoline-pyrrolidine derivatives and other cell wall-targeting fused heterocyclic derivatives. Interference with membrane function was also commonly reported and was associated with alterations in ergosterol biosynthesis, increased membrane permeability, and loss of cellular integrity. These effects were particularly associated with tetrazole- and dispiro-based scaffolds. Antibiofilm and anti-virulence effects represented another important mechanism, which include multiple factors such as inhibition of biofilm formation, suppression of hyphal transition, and reduction in adhesion capacity (Figure 2). These effects were most prominent in spirooxindole and dispiro derivatives. A smaller number of investigations reported oxidative stress induction and apoptosis-like cell death, indicated by increased reactive oxygen species (ROS) levels and downstream cellular damage. However, these findings remain limited to fewer experimental models (Table 3).

Molecular docking and in silico studies suggested potential interactions between pyrrolidine derivatives and fungal enzymes involved in cell wall synthesis and sterol metabolism. While computational predictions provide valuable insights, their findings must be validated experimentally and should be interpreted as supporting evidence, rather than conclusive. Collectively, pyrrolidine-based derivatives exhibit a multitarget antifungal profile involving cell wall disruption, membrane instability, and suppression of virulence mechanisms.

### 3.5. Quality of the Included Study

The methodological quality assessment of the included studies is summarized in Table 4. Overall, most studies demonstrated moderate to high methodological quality based on the predefined appraisal criteria, although variability in experimental standardization and statistical reporting was observed across investigations.

All included studies clearly described study objectives, experimental design, pyrrolidine derivative characteristics, target *Candida* species, methodologies, and primary outcomes. A small number of studies lacked detailed statistical analysis or did not clearly report quantitative measurements, while a few studies did not explicitly mention comparator or control conditions.

The quality assessment scores of the included studies ranged from seven to nine out of a maximum score of nine, reflecting moderate to high methodological rigour overall. However, variations in experimental designs and outcome assessment methods were evident among the studies.

The methodological quality of each included study was additionally summarized using a total quality score obtained from the nine appraisal criteria. Studies were categorized according to their total scores as high-quality (8–9 points), moderate-quality (6–7 points), or low-quality (≤5 points).

The quality assessment demonstrated that the included studies had overall moderate-to-high methodological quality, with total scores ranging from seven to nine out of nine. Among the 12 included studies, nine were classified as high-quality (scores 8–9), whereas three studies were categorized as moderate-quality (scores 6–7). No study was rated as low-quality.

### 3.6. Risk-of-Bias Assessment

**Figure 3:**
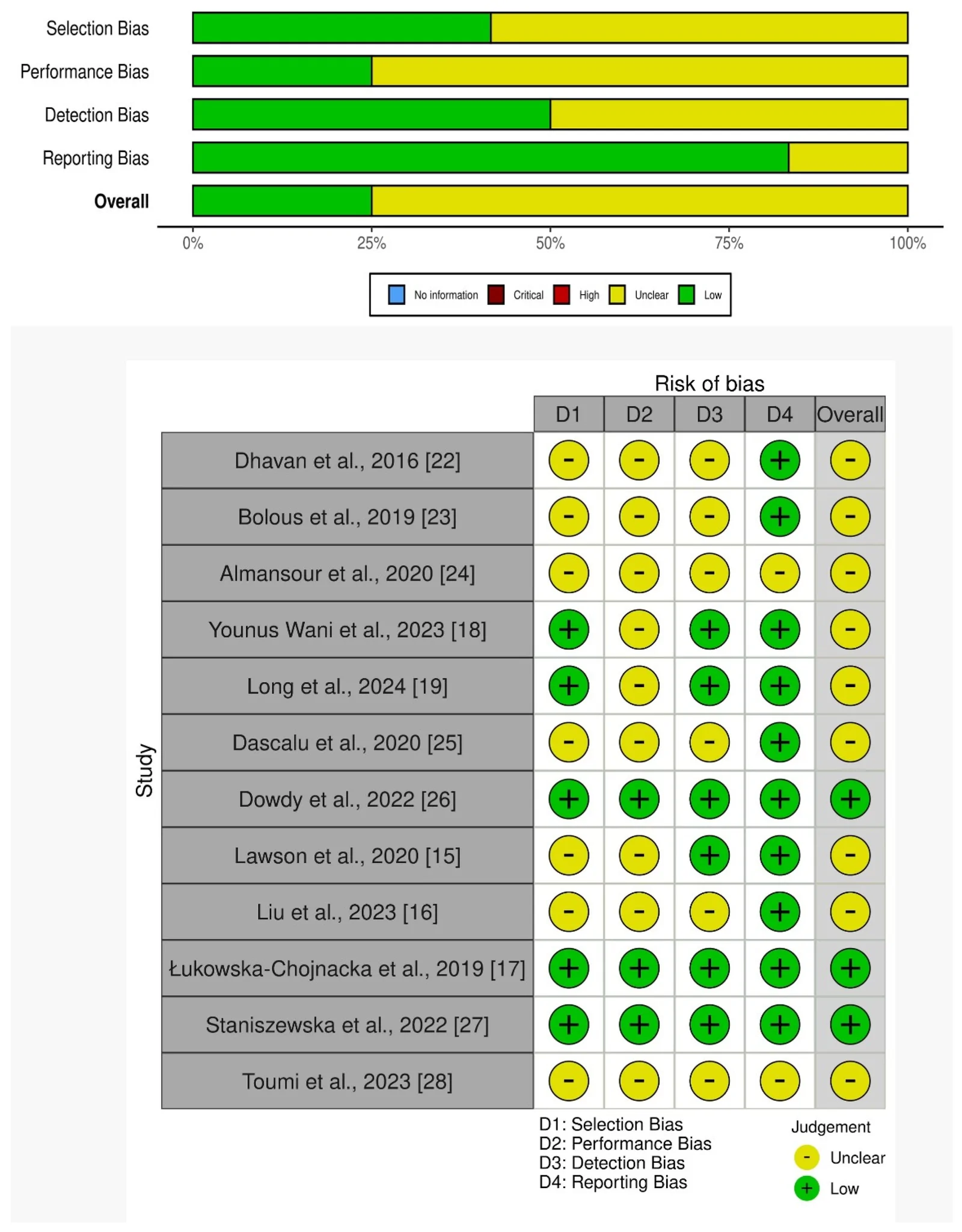
Risk-of-bias summary and graphical representation of included studies. Green indicates low risk of bias, yellow indicates unclear risk of bias, and red indicates high risk of bias [15,16,17,18,19,22,23,24,25,26,27,28].

The result of the risk-of-bias evaluation is reported in Figure 3. Generally, most studies that were included in this review showed a low to moderate risk of bias within each assessed domain. Most studies described the aim of experiments, interventions, outcomes, and methodology properly.

However, several studies demonstrated unclear risk of bias within domains such as comparator standardization, blinding outcome measure assessment, and methodological reporting. Differences in experimental design and reporting led to heterogeneity of methodology quality.

Risk-of-bias visualization was performed using the robvis tool to generate traffic-light and summary plots for interpretation of methodological consistency across included studies.

## 4. Discussion

The present systematic review summarizes evidence from 12 experimental studies investigating pyrrolidine-based derivatives as potential antifungal agents against *Candida* species. Collectively, the available evidence suggests that pyrrolidine-containing compounds represent a structurally diverse class of molecules with promising antifungal activity. Beyond inhibiting fungal growth, several derivatives also interfere with key virulence traits, including biofilm formation, hyphal transition, and fungal adhesion, suggesting potential advantages over conventional antifungal agents that primarily target fungal viability [3,16,17,19,26,29].

A consistent finding across the included studies was the influence of structural modification on antifungal activity. Among the included studies, spiro-fused pyrrolidine derivatives, particularly spirooxindole and dispiro scaffolds, exhibited potent antifungal activity [15,23,26,29,30]. Their enhanced activity may be attributed to increased molecular rigidity and three-dimensional architecture, which may improve interactions with fungal targets and enhance binding affinity, as suggested by experimental and computational studies. The synthetic versatility and conformational flexibility of related heterocyclic antifungal scaffolds further facilitate structural optimization, making pyrrolidine-containing scaffolds attractive platforms for the development of biologically active molecules [16,19,21,30,31].

Comparative analysis further indicated that heterocycle-fused pyrrolidines incorporating triazole, quinoline, and tetrazole moieties frequently demonstrated more potent antifungal activity in individual studies than simple pyrrolidine scaffolds, highlighting the importance of scaffold complexity and heterocyclic fusion in optimizing biological activity. The importance of heterocyclic scaffold diversity is also reflected in modern drug discovery, where heterocycles constitute a major proportion of approved therapeutic agents because of their ability to optimize target affinity, pharmacokinetic properties, and molecular diversity [16,17,18,29,32]. However, differences in assay methods, *Candida* strains, and experimental conditions limit direct comparison between studies and limit confidence in establishing definitive structure–activity relationships (SARs). Therefore, the proposed SAR trends should be regarded as preliminary until validated through standardized comparative investigations.

Mechanistic investigations further demonstrated a close relationship between chemical structure and antifungal mode of action. Spiro-fused derivatives showed membrane-related effects and antibiofilm activity; their enhanced activity has been proposed to be associated with increased structural rigidity and three-dimensional organization [16,26,27]. In contrast, heterocycle-fused derivatives, particularly triazole- and quinoline-containing compounds, predominantly targeted enzymes involved in cell wall biosynthesis and ergosterol metabolism. This finding is consistent with previous reports demonstrating that inhibition of sterol 14α-demethylase (CYP51), a key enzyme in ergosterol biosynthesis, remains one of the most successful strategies for antifungal drug development and can be effectively explored through structure-based molecular modelling approaches [33].

Tetrazole-containing derivatives demonstrated effects on fungal adhesion and virulence-associated processes, while molecular interactions proposed from computational studies require further experimental validation [17,18,27]. Among all scaffold classes evaluated, spirooxindole derivatives represent one of the more extensively investigated and promising scaffold classes because they consistently demonstrated low MIC values, antibiofilm activity, and acceptable cytotoxicity profiles [16,23,26,28]. The ability of several pyrrolidine derivatives to simultaneously inhibit fungal growth and attenuate virulence factors, including biofilm formation and yeast-to-hyphae transition, represents a potentially important therapeutic advantage [16,17,18,26,29]. Targeting pathogenicity alongside fungal viability may improve treatment outcomes and potentially reduce the selective pressure associated with conventional fungicidal therapies; however, these potential benefits require validation in appropriate preclinical and clinical studies.

An additional important observation from the included studies is that several pyrrolidine derivatives show activity against drug-resistant clinical isolates, including multidrug-resistant *Candida auris*. Their reported activity against resistant isolates highlights their potential; however, whether multitarget activity translates into reduced resistance emergence remains to be experimentally established [3,8,12]. Nevertheless, these findings are derived predominantly from in vitro investigations, and their clinical significance remains to be established through comprehensive preclinical evaluation.

Despite the encouraging antifungal activity reported across the included studies, several limitations of the current evidence should be acknowledged. Only 12 studies fulfilled the predefined eligibility criteria, reflecting the limited literature specifically investigating pyrrolidine-based compounds against *Candida* species. Furthermore, considerable methodological heterogeneity including differences in *Candida* strains, antifungal susceptibility assays, experimental conditions, and outcome measurements precluded quantitative synthesis and limited direct comparison of antifungal efficacy. In addition, only a limited number of studies comprehensively evaluated toxicity, pharmacokinetics, mechanism of action, or in vivo efficacy, thereby limiting the translational applicability of the currently available evidence. Toxicological evidence remains limited because only a minority of studies evaluated cytotoxicity and no long-term toxicity studies were available.

Future investigations should focus on rational optimization of pyrrolidine scaffolds by integrating SAR findings with mechanistic insights. Similar SAR-guided optimization strategies have successfully improved the biological activity of several heterocyclic scaffold classes, highlighting the value of systematic structural modification during lead optimization [34]. Structural modification through rigid spiro frameworks and heterocyclic pharmacophores may further enhance antifungal potency, target specificity, and antibiofilm activity [16,17,18,19,21,28,30]. Standardized antifungal susceptibility testing, detailed mechanistic evaluation, pharmacokinetic profiling, toxicity assessment, and validation in appropriate animal models will be essential for translating these promising compounds into clinically relevant antifungal therapies [8,12]. In addition, computational simulation approaches should be increasingly integrated into future antifungal drug discovery pipelines. Techniques such as molecular dynamics simulations can provide detailed insights into the stability of ligand–target interactions beyond conventional molecular docking, whereas binding free-energy calculations may improve the prediction of binding affinity toward fungal targets [28,35,36]. Furthermore, pharmacophore modelling, quantitative structure–activity relationship (QSAR) analysis, virtual screening, and in silico ADMET prediction can facilitate the identification and optimization of promising pyrrolidine derivatives prior to experimental evaluation [35,36]. These approaches may facilitate prioritization and optimization of candidate molecules before experimental validation.

Overall, pyrrolidine derivatives represent promising lead scaffolds for antifungal drug discovery against *Candida* species. Their structural diversity, multitarget mechanisms of action, and ability to interfere with important fungal virulence factors support continued investigation of this chemical class [3,12,13]. However, the current evidence remains limited by the small number of available studies and the lack of comprehensive preclinical evaluation. Further standardized studies are required to validate the proposed SAR trends and facilitate the development of effective pyrrolidine-based antifungal agents against *Candida* species.

## 5. Conclusions

Pyrrolidine derivatives have emerged as potential structural frameworks in the field of antifungal drugs, demonstrating potential antifungal activity against *Candida* species, including resistant strains. The antifungal action of the reviewed molecules was reported through several different mechanisms such as disruption of the cell wall or membrane integrity, inhibition of biofilms and modulation of virulence-associated pathways. Structural modifications, including spiro-fused and heterocycle-containing derivatives, were associated with improved antifungal potency.

The number of available in vivo studies remains limited when it comes to validating these results and comprehensive pharmacological and toxicological evaluations remain scarce to date. Standardizations of experimental methodologies and structure–activity relationship optimization are needed for more effective comparisons between the studies. Although this review identifies pyrrolidine derivatives as antifungal scaffolds, the strength of the conclusions is constrained by the relatively small number of eligible studies (*n* = 12), methodological heterogeneity, and the inability to perform a quantitative meta-analysis. Nevertheless, the available evidence consistently supports further preclinical investigation of these compounds.

Future studies should integrate advanced computational simulation approaches, including molecular dynamics simulations, virtual screening, QSAR modelling, and in silico ADMET prediction, with experimental validation to accelerate the rational design and optimization of pyrrolidine-based antifungal agents [35,36].

In summary, pyrrolidine derivatives represent a promising platform for the development of active antifungal drugs. However, more preclinical studies are needed before advancing towards the clinic.

## Figures and Tables

**Table 1:** Characteristics of included studies on pyrrolidine-based anticandidal agents.

Author and Reported Year	Study Location	Study Design	Compounds Tested	Targeted Organism	Methodology	Antifungal Potency	Key Findings	Cytotoxicity/Safety
**Dhavan et al., 2016 [22]**	Italy	In vitro	2,3-Pyrrolidinedione derivatives	*C. albicans*, *Streptococcus mutans* (clinical isolates)	Antimicrobial and biofilm inhibition assay	Comparable to chlorhexidine (biofilm inhibition)	Antibiofilm activity against oral pathogens	Not reported
**Bolous et al., 2019 [23]**	United States	In vitro	Spirooxindole–pyrrolidine hybrids	*C. albicans*, *Cryptococcus *(clinical isolates)	MIC, biofilm inhibition assay	MIC: 4–16 µg/mL	Biofilm and hyphal inhibition	Not reported
**Almansour et al., 2020 [24]**	Saudi Arabia	In vitro	Dispirooxindolopyrrolidine hybrids	*C. albicans*, *Aspergillus niger*, Bacteria.	Agar diffusion, MIC	Significant antimicrobial activity	Broad-spectrum activity against fungi and bacteria	Not reported
**Younus Wani et al., 2023 [18]**	Saudi Arabia, South Africa	In vitro	Pyrrolidine–triazole derivatives	*C. auris (MRL6057)*	MIC, MFC, cell viability assay	MIC ~0.977 µg/mL (lead compound)	Antifungal + cell cycle arrest	Minimal hemolysis
**Long et al., 2024 [19]**	China	In vitro, in silico	Spiroquinoline–pyrrolidinediones	*C. albicans (ATCC 76615),* *Aspergillus flavus (ATCC 16870),* *Aspergillus fumigatus (GIMCC 3.19),* *Cryptococcus neoformans (ATCC 32719)*	MIC, chitin synthase inhibition assay, sorbitol protection assay, cytotoxicity assay, molecular docking	Potent antifungal activity	Cell wall inhibition	Low cytotoxicity
**Dascalu et al., 2020 [25]**	France, Romania	In vitro	Hydrazine/acyl hydrazone pyrrolidones	Multiple fungi incl. *Candida* sp.	MIC, cytotoxicity assay	MIC_50_ lower than standard drugs (some compounds)	Broad antifungal spectrum	Low cytotoxicity
**Dowdy et al., 2022 [26]**	United States	In vitro, in vivo	Spirooxindole–pyrrolidine	*C. albicans* (*ATCC 10231* and clinical isolate), *Cryptococcus neoformans* (clinical isolate)	MIC, cytotoxicity assay, hyphal and biofilm assay	Superior antibiofilm activity compared with FDA drugs in biofilm assay	Antibiofilm + non-toxic	Non-toxic in vitro
**Lawson et al., 2020 [15]**	United States	In vitro	Dispiropyrrolidine hybrids	*C. albicans (ATCC10231), Cryptococcus (clinical strains)*	MIC, hyphal and biofilm assay, cytotoxicity assay	MIC: 0.5–8 µg/mL	Activity against resistant strain activity	No toxicity reported
**Liu et al., 2023 [16]**	China	In vitro, in silico	Spiroquinoline–pyrrolidine derivatives	*C. albicans (ATCC 76615), Aspergillus flavus (ATCC 16870),* *Aspergillus fumigatus (GIMCC 3.19),* *Cryptococcus neoformans (ATCC 32719)*	MIC, sorbitol protection assay, drug combination assay, molecular docking	Potent antifungal activity	Cell wall stress response	Not reported
**Łukowska-Chojnacka et al., 2019 [17]**	Poland	In vitro, in vivo	Tetrazole–pyrrolidine scaffold	*C. albicans (ATCC90028)*	MIC, ergosterol assay, antibiofilm assay, cell adhesion inhibition, flow cytometry, confocal laser scanning microscopy	Effective MIC-based inhibition	Anti-virulence	Low toxicity (Vero cells)
**Staniszewska et al., 2022 [27]**	Poland	In vitro, in vivo	Tetrazole–benzodiazepine–pyrrolidine	*C. albicans (SC5314)*	MFC, flow cytometry, cytotoxicity assay, histopathological assay, ergosterol biosynthesis, gene expression	Reduced virulence, gene expression modulation	Anti-pathogenic strategy	Non-toxic
**Toumi et al., 2023 [28]**	Tunisia	In vitro, in vivo, In silico	Spiropyrrolidine-fused hydantoin/spirooxindole hybrids	*C. albicans (ATCC 90028)* and *C. krusei (ATCC 6258)*	Agar microdilution assay, MIC determination, MFC determination, SAR evaluation, molecular docking	Highest activity against C. albicans (MFC 23.43 µg/mL) and C. krusei (MFC 46.87 µg/mL); most derivatives exhibited fungicidal activity	Potent anticandidal activity	Not tested

**Table 2:** Structure–activity relationship (SAR) summary.

Scaffold Class	Scaffold Structure	Key Structural Feature	Effect on Activity	SAR Interpretation	References
Simple pyrrolidines, pyrrolidinediones	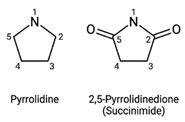	Pyrrolidine core (basic scaffold)	Moderate to strong antifungal activity	Confers baseline antifungal pharmacophore, essential heterocyclic core for activity	Dhavan et al., 2016 [22]
Spirooxindole–pyrrolidine, dispiro systems	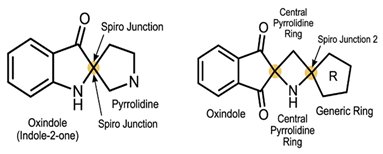	Spiro fusion	Increased potency (low MIC)	Spiro fusion may increase 3D rigidity, enhancing target binding and selectivity	Bolous et al., 2019 [23]
Dispiropyrrolidine hybrids	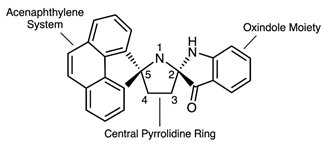	Dispiro architecture	Increased broad-spectrum activity	Increased conformational constraint improves membrane and enzyme interactions	Lawson et al., 2020 [15]
Pyrrolidine–triazole derivatives	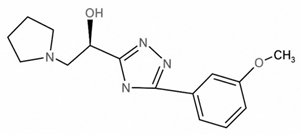	Triazole-linked hybrids	Increased activity vs. *C. auris*	Triazole moiety enhances interaction with fungal enzymes and improves antifungal spectrum	Younus Wani et al., 2023 [18]
Tetrazole–pyrrolidine	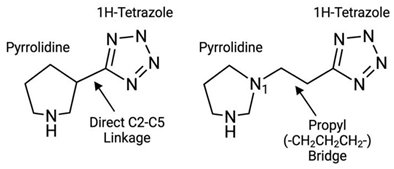	Tetrazole group	Increased anti-virulence effect	Tetrazole may increase hydrogen bonding and improve binding to sterol biosynthesis targets	Łukowska-Chojnacka et al., 2019 [17]; Staniszewska et al., 2022 [27]
Spiroquinoline–pyrrolidine	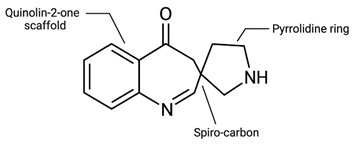	Quinoline fusion	Increased cell wall inhibition	Aromatic quinoline may enhance π–π stacking with enzymatic active sites	Liu et al., 2023 [16]
Pyrrolidone hydrazones	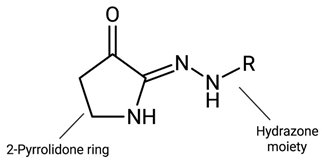	Hydrazone group	Increased broad-antifungal-spectrum	Flexible linker may improve target adaptability and enzyme binding diversity	Dascalu et al., 2020 [25]
Phenyl derivatives	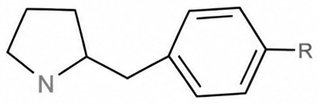	Aromatic substitution	Variable potency	Electronic and steric effects strongly influence antifungal potency	Dowdy et al., 2022 [26]

**Table 3:** Mechanisms of action and anti-virulence effects.

Scaffold Class	Reference Drugs	Mechanism of Action	Experimental Evidence	Biological Outcome	References
**Spiroquinoline–pyrrolidine**	Fluconazole, Polyoxin B, Micafungin	Cell wall inhibition (chitin synthase)	Sorbitol protection assay, molecular docking	Cell wall stress and growth inhibition	Liu et al., 2023 [16], Long et al., 2024 [19]
**Tetrazole–pyrrolidine**	Amphotericin B	Ergosterol biosynthesis disruption	Ergosterol quantification	Membrane instability	Łukowska-Chojnacka et al., 2019 [17]; Staniszewska et al., 2022 [27]
**Dispiropyrrolidine hybrids**	Fluconazole	Membrane disruption	PI uptake assay	Loss of cell integrity	Lawson et al., 2020 [15]
**Spirooxindole derivatives**	Ketoconazole	Biofilm inhibition	Crystal violet assay	Reduced biofilm biomass	Dowdy et al., 2022 [26]
**Spirooxindole hybrids**	Fluconazole, Flucytosine	Hyphal suppression	Morphogenesis assay	Reduced virulence	Almansour et al., 2020 [24]; Bolous et al., 2019 [23]
**Dispiropyrrolidine derivatives**	Fluconazole	Oxidative stress induction	ROS detection assay	Apoptosis-like death	Lawson et al., 2020 [15]
**Pyrrolidine–triazoles**	Amphotericin B	Apoptosis induction	Flow cytometry	Cell cycle arrest	Younus Wani et al., 2023 [18]
**Tetrazole derivatives**	Amphotericin B	Adhesion inhibition	Adhesion assay	Reduced colonization	Łukowska-Chojnacka et al., 2019 [17]
**Tetrazole–benzodiazepine hybrids**	Amphotericin B	Gene expression modulation	RT-qPCR	Virulence gene suppression	Staniszewska et al., 2022 [27]

**Table 4:** Quality assessment and methodological rating of the studies included in the systematic review.

Quality Appraisal Criteria	Dhavan et al., 2016 [22]	Bolous et al., 2019 [23]	Almansour et al., 2020 [24]	Younus Wani et al., 2023 [18]	Long et al., 2024 [19]	Dascalu et al., 2020 [25]	Dowdy et al., 2022 [26]	Lawson et al., 2020 [15]	Liu et al., 2023 [16]	Łukowska-Chojnacka et al., 2019 [17]	Staniszewska et al., 2022 [27]	Toumi et al., 2023 [28]
1. Was the study objective clearly mentioned?	1	1	1	1	1	1	1	1	1	1	1	1
2. Was the study design (in vitro, in vivo, or in silico) clearly described?	0	1	1	0	1	0	0	0	1	0	0	1
3. Were the selection criteria clear?	0	1	1	1	1	1	1	1	1	1	1	1
4. Were the inclusion and exclusion criteria properly stated?	1	1	1	1	1	1	1	1	1	1	1	0
5. Was the pyrrolidine derivative used clearly mentioned?	1	1	1	1	1	1	1	1	1	1	0	1
6. Was the organism used clearly mentioned?	1	1	1	1	1	1	1	1	1	1	1	1
7. Was the methodology clearly described and specific?	1	1	1	1	1	0	1	1	1	1	1	1
8. Was the control group included in the study?	1	1	1	1	1	1	1	1	1	1	1	1
9. Were the outcomes clearly reported?	1	1	1	1	1	1	1	1	1	1	1	1
Total score (/9)	7	9	9	8	9	7	8	8	9	8	7	8
Quality rating	Moderate	High	High	High	High	Moderate	High	High	High	High	Moderate	High

Each criterion was scored as “1” when adequately reported and “0” when insufficiently reported or not available. The total quality score was calculated by summing the scores of all nine criteria (maximum score = 9). The overall quality ratings were classified as high-quality (8–9 points), moderate-quality (6–7 points), and low-quality (≤5 points).

## Data Availability

The original contributions generated for this study are included in the article. Further inquiries may be addressed to the corresponding author.

## Abbreviations

The following abbreviations are used in this manuscript:

SAR	Structure–activity relationship
PRISMA	Preferred Reporting Items for Systematic Review and Meta-Analyses
MIC	Minimum inhibitory concentration
MFC	Minimum fungicidal concentration
